# Stachydrine Hydrochloride Suppresses Hepatocellular Carcinoma Progression by Reprogramming Macrophage Polarization Through the LIF/PI3K/Akt Axis

**DOI:** 10.3390/ijms27219911

**Published:** 2026-11-05

**Authors:** Xiaomei Bao, Ye Zhang, Lingjuan Sun, Chunlong He, Dandan Li, Xinran Shi, Yuehong Ma

**Affiliations:** 1 School of Pharmacy, Inner Mongolia Medical University, Hohhot 010110, China; 20060152@immu.edu.cn (X.B.); 20120128@immu.edu.cn (Y.Z.); 2025110132@stu.immu.edu.cn (L.S.); 20080264@immu.edu.cn (C.H.); 2026110116@stu.immu.edu.cn (D.L.); 2 Baotou Clinical Medical College, Inner Mongolia Medical University, Baotou 014040, China; 2023041732@stu.immu.edu.cn; 3 School of Basic Medicine, Inner Mongolia Medical University, Hohhot 010010, China

**Keywords:** stachydrine hydrochloride, hepatocellular carcinoma, tumor-associated macrophages, M2 macrophages, LIF/PI3K/Akt signaling pathway

## Abstract

Hepatocellular carcinoma (HCC) carries a poor prognosis, largely owing to an immunosuppressive tumor immune microenvironment (TIME) shaped by M2-polarized tumor-associated macrophages (TAMs). Stachydrine hydrochloride (SH), an alkaloid derived from *Panzeria alashanica* Kupr., exerts anti-inflammatory and anti-tumor effects; however, its immunomodulatory action in HCC remains unclear. Here, we investigated whether SH restrains HCC progression by regulating TAM polarization through the LIF/PI3K/AKT pathway, using RAW264.7, THP-1, HepG2, and Huh-7 cells; a subcutaneous tumor model; and bioinformatic analysis of clinical samples. SH modulated macrophage phenotypes dose-dependently without obvious cytotoxicity, promoting M1 polarization while suppressing M2 polarization and autophagy in M2 macrophages, and enhanced macrophage phagocytosis of HCC cells. Conditioned medium from SH-treated M2 macrophages inhibited HCC cell proliferation, migration, and epithelial–mesenchymal transition. Mechanistically, SH suppressed LIF/PI3K/AKT activation in M2 macrophages, and LIF overexpression showed a trending capacity to partially counteract SH-mediated M2-suppressive effects. Bioinformatics linked high M2-TAM infiltration with poor HCC prognosis. In vivo, SH retarded tumor growth, decreased intratumoral M2-TAM infiltration, and reduced Ki-67 levels. Collectively, SH remodels the TIME to restrain HCC progression, and these effects are associated with inhibition of M2-TAM polarization and attenuation of LIF/PI3K/Akt signaling, revealing a novel immunotherapeutic mechanism and supporting SH as a promising natural agent for HCC immunotherapy.

## 1. Introduction

Hepatocellular carcinoma (HCC) is the third leading cause of cancer-related mortality worldwide, characterized by limited therapeutic options and a 5-year survival rate of less than 20% [1]. The tumor immune microenvironment (TIME) plays a pivotal role in HCC progression, with tumor-associated macrophages (TAMs) representing the most abundant immune cell population [2]. In the context of HCC, TAMs predominantly exhibit an M2-polarized phenotype, which promotes tumor growth, angiogenesis, immune evasion, and metastasis [3,4]. Increasing studies have evidenced that the immunosuppressive tumor microenvironment (TME) is responsible for such insufficient response to immunotherapy [5]. Immunotherapeutic strategies centered on the modulation of tumor-associated macrophages (TAMs) have emerged as a rapidly expanding research focus in the management of hepatocellular carcinoma (HCC). Representative approaches include impeding the chemotactic recruitment of monocytes into the HCC tumor microenvironment (TME), depleting TAMs within tumor tissues, redirecting TAM polarization from the pro-tumor M2 phenotype toward the anti-tumor M1 phenotype, augmenting the phagocytic and tumoricidal capacity of macrophages, and neutralizing pro-tumor mediators secreted by TAMs [6,7,8]. M2-polarized TAMs produce a wide array of immunosuppressive and oncogenic cytokines, such as transforming growth factor-β (TGF-β), interleukin-10 (IL-10), and leukemia inhibitory factor (LIF). These factors act in concert to promote HCC cell proliferation, invasion, epithelial–mesenchymal transition (EMT), and immune evasion while concurrently dampening anti-tumor immune responses [9,10]. Therefore, therapeutic interventions that suppress M2-type TAM polarization or interrupt its downstream signaling pathways represent a highly promising avenue for improving HCC immunotherapy.

Macrophages exhibit remarkable phenotypic plasticity. In response to distinct microenvironmental signals, macrophages can acquire classically activated M1-like or alternatively activated M2-like phenotypes. M1-like macrophages are commonly characterized by the expression of inflammatory and anti-tumor markers, including TNF-α, IL-6, iNOS and CD86. By contrast, M2-like macrophages express markers such as TGF-β, IL-10, Arg-1 and CD206 and generally contribute to tissue remodeling, immune suppression and tumor progression [11,12]. Thus, strategies that inhibit M2 polarization or reprogram macrophages toward an M1-like phenotype represent a promising approach for HCC therapy.

**Figure 1:**
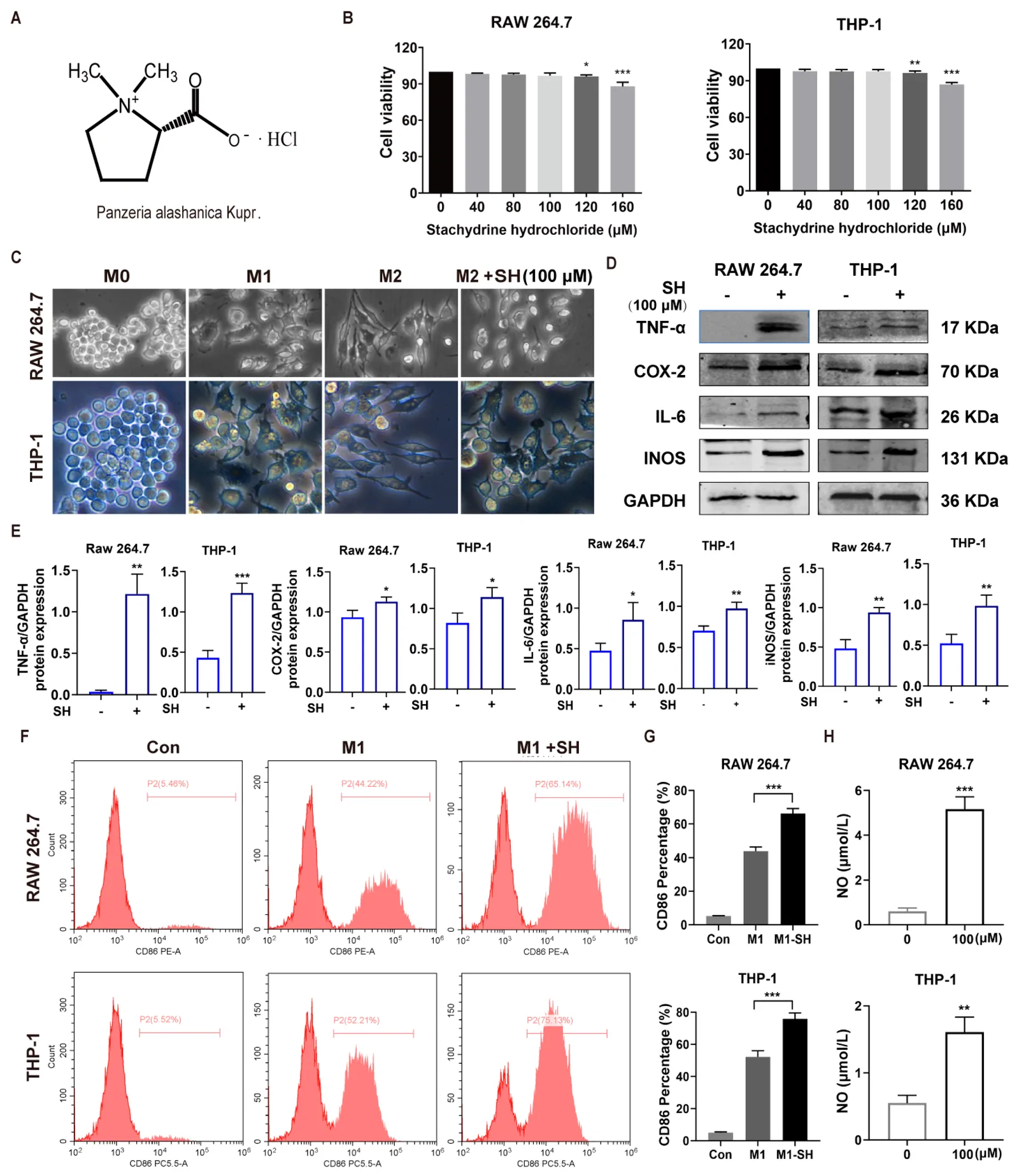
SH promotes M1−like polarization. (**A**) Chemical structure of stachydrine hydrochloride (SH). (**B**) Cell viability of RAW 264.7 and THP-1 cells treated with 0–160 μM SH. (**C**) Representative morphological images of RAW264.7 and THP-1 macrophages under M0, M1, M2 and M2 + SH treatment conditions. M2-polarized macrophages were used as the reference for morphological comparison with the M2 + SH group. (**D**,**E**) Western blot analysis of M1 markers (TNF-α, COX-2, IL-6, iNOS) in M1-polarized macrophages. (**F**,**G**) Flow cytometry analysis and quantification of CD86-positive macrophages. (**H**) NO production after SH treatment in M1 macrophages. Data are presented as mean ± SD (*n* = 3). * *p* < 0.05; ** *p* < 0.01; *** *p* < 0.001.

Stachydrine hydrochloride (SH), a quaternary ammonium alkaloid isolated from *Panzeria alashanica* Kupr. (Figure 1A), has been reported to exert diverse pharmacological activities, including anti-inflammatory, cardioprotective, and anti-tumor effects across multiple cancer types [13,14]. Despite these findings, the role of SH in regulating TAM polarization and its subsequent impact on HCC progression remains entirely unexplored.

Leukemia inhibitory factor (LIF) was initially cloned as a differentiation inducer and proliferation inhibitor in M1 myeloid leukemia cells. However, increasing evidence has established LIF as an oncogenic mediator across a broad spectrum of solid tumors [15,16,17]. Concurrently, the PI3K/Akt signaling axis has been recognized as a central intracellular driver that orchestrates macrophage polarization and functional reprogramming within the hepatocellular carcinoma (HCC) tumor immune microenvironment [18,19]. Moreover, LIF remodels the tumor immune microenvironment, prominently modulating macrophage polarization to establish an immunosuppressive milieu that facilitates tumor progression, largely via activation of the JAK/STAT3 and PI3K/AKT signaling cascades [20]. Functionally, M2-polarized tumor-associated macrophages (TAMs) activated by PI3K/Akt signaling secrete abundant pro-tumor mediators to facilitate HCC cell proliferation, epithelial–mesenchymal transition (EMT), invasion and immune escape, ultimately accelerating malignant progression of HCC and correlating with poor clinical prognosis of patients [19,21]. In the present study, we investigated the effect of SH on M2 TAM polarization both in vitro and in vivo and further delineated the underlying molecular mechanisms. Our findings may provide a novel immunotherapeutic strategy for HCC by targeting the LIF/PI3K/AKT axis via a natural alkaloid compound.

## 2. Results

### 2.1. SH Promotes M1-like Polarization

The chemical structure of SH and its botanical source are shown in Figure 1A. To determine a suitable experimental concentration, RAW264.7 and THP-1 macrophages were exposed to increasing concentrations of SH. SH at 40, 80 and 100 μM did not markedly reduce cell viability, whereas 120 and 160 μM caused a significant decrease in cell viability, especially at 160 μM (Figure 1B). Therefore, 100 μM SH was selected as a relatively safe concentration for subsequent functional experiments. We then polarized macrophages to the M0, M1, and M2 phenotypes and observed that SH treatment altered macrophage morphology (Figure 1C). Western blotting confirmed that SH increased the protein levels of M1-associated inflammatory markers, including TNF-α, COX-2, IL-6 and iNOS (Figure 1D,E). Flow cytometry further demonstrated that SH increased the CD86-positive macrophage population (Figure 1F,G). SH also significantly increased nitric oxide (NO) production in both macrophage models (Figure 1H). These results indicate that SH promotes M1-like macrophage activation.

### 2.2. SH Suppressed M2 Macrophage Polarization

**Figure 2:**
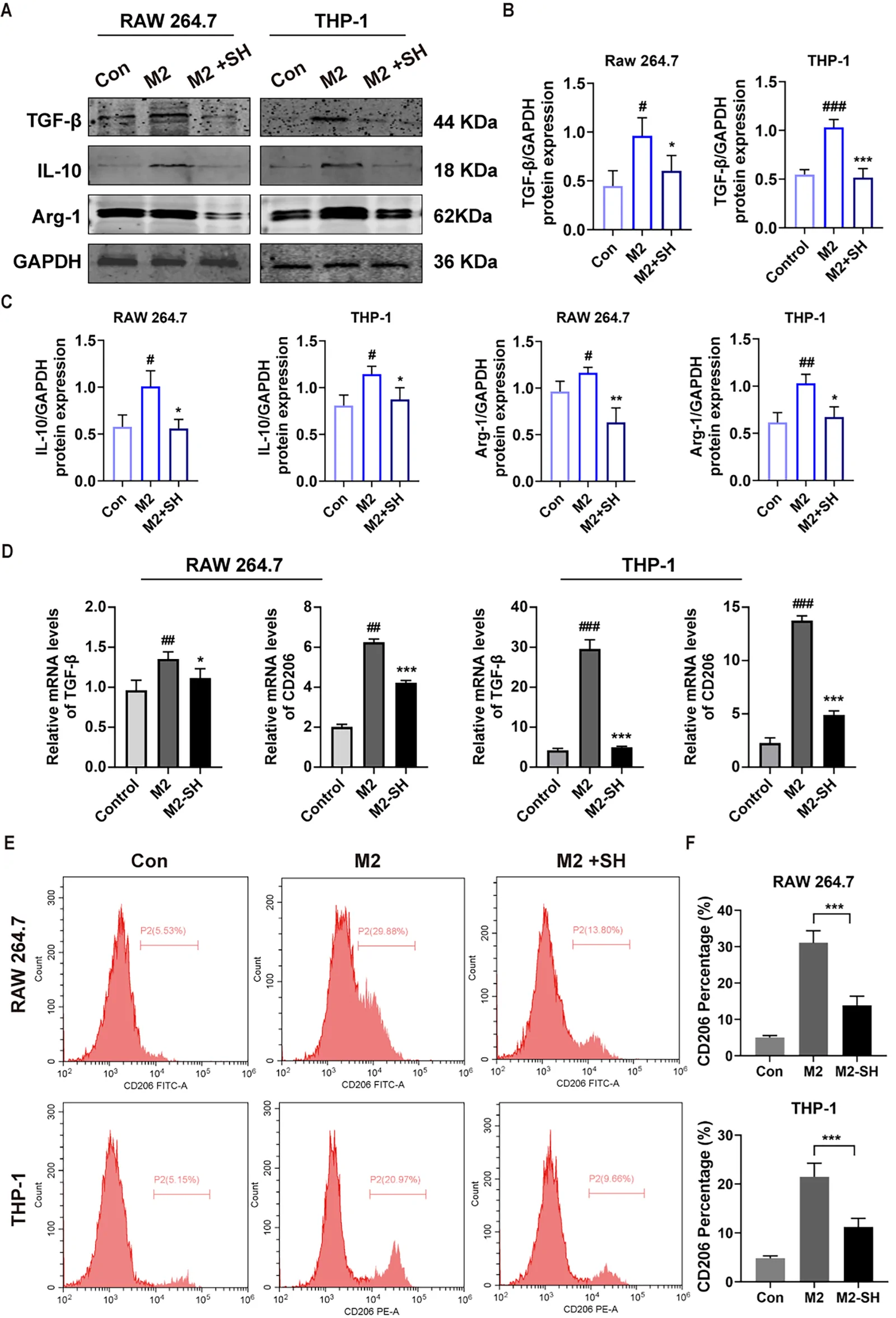
SH suppressed M2 macrophage polarization. (**A**–**C**) Western blot analysis of M2-associated proteins TGF-β, IL-10 and Arg-1 in RAW264.7 and THP-1 cells. (**D**) Relative mRNA expression levels of TGF-β and CD206 in RAW264.7 and THP-1 cells. (**E**,**F**) Flow cytometry analysis and quantification of CD206-positive macrophages. Data are presented as mean ± SD (*n* = 3). # *p* < 0.05, ## *p* < 0.01 and ### *p* < 0.001 versus Control; * *p* < 0.05, ** *p* < 0.01 and *** *p* < 0.001 versus M2.

The effect of SH on M2 polarization was next examined. Western blotting showed that M2 induction increased the expression of TGF-β, IL-10 and Arg-1 in both RAW264.7 and THP-1 cells. SH treatment reduced these M2-associated proteins, indicating that SH inhibited the formation of an M2-like macrophage phenotype (Figure 2A–C). Consistently, the mRNA levels of TGF-β and CD206 were markedly increased after M2 induction but were significantly decreased by SH treatment (Figure 2D). Flow cytometry further confirmed that SH reduced the CD206-positive macrophage population (Figure 2E,F). These findings demonstrate that SH suppresses M2 macrophage polarization.

### 2.3. SH Inhibited M2-Associated Autophagy and Enhanced Macrophage Phagocytosis of HCC Cells

**Figure 3:**
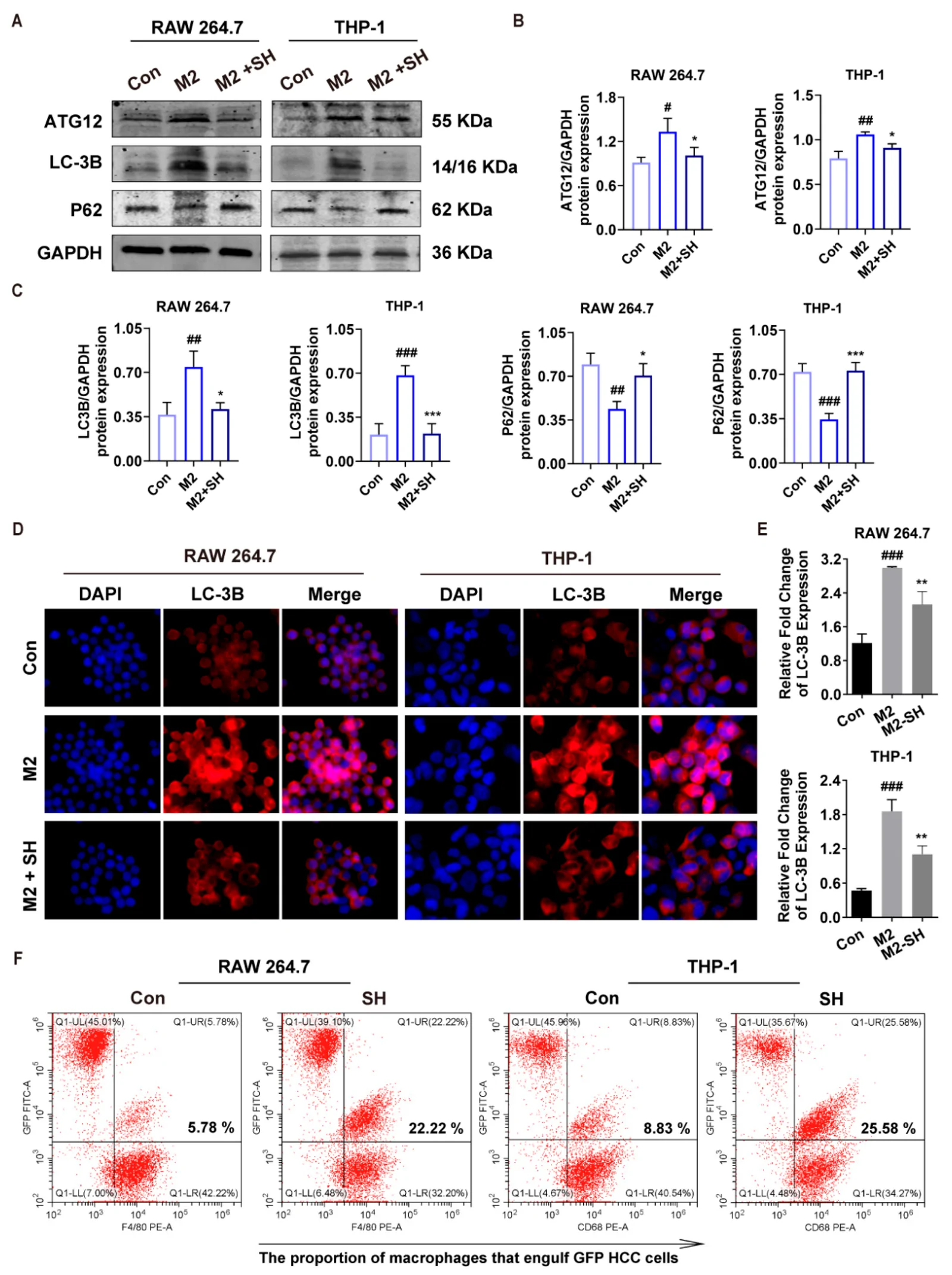
SH inhibited M2-associated autophagy and enhanced macrophage phagocytosis of HCC cells. (**A**–**C**) Western blot analysis of autophagy-related proteins (ATG12, LC3B, p62) in RAW264.7 and THP-1 cells. (**D**) Immunofluorescence staining of LC3B. DAPI (blue) indicates nuclei; LC3B is stained red. Merge shows the merged fluorescence channels. (**E**) Quantification of LC3B expression. (**F**) Flow cytometry analysis of macrophages engulfing GFP-labeled HCC cells. Data are presented as mean ± SD (*n* = 3). ### *p* < 0.001, ## *p* < 0.01 and # *p* < 0.05 versus Control; *** *p* < 0.001, ** *p* < 0.01 and * *p* < 0.05 versus M2.

Because autophagy is involved in macrophage functional remodeling, autophagy-related markers were detected. M2 induction increased ATG12 and LC3B expression, whereas SH treatment reduced ATG12 and LC3B and restored P62 expression in RAW264.7 and THP-1 cells (Figure 3A–C). Immunofluorescence staining confirmed that LC3B fluorescence intensity was enhanced in M2 macrophages and reduced after SH treatment (Figure 3D). Quantitative analysis showed that SH significantly decreased LC3B expression compared with the M2 group (Figure 3E). These data suggest that SH inhibits M2-associated autophagy activation.

To further evaluate macrophage anti-tumor function, a phagocytosis assay was performed using GFP-labeled HCC cells (Huh-7-EGFP). SH treatment markedly increased the proportion of macrophages engulfing GFP-positive tumor cells. In RAW264.7 cells, the phagocytic proportion increased from 5.78% to 22.22%, while in THP-1 cells it increased from 8.83% to 25.58% (Figure 3F). Therefore, SH not only suppresses M2 polarization but also enhances macrophage-mediated clearance of HCC cells.

### 2.4. SH-Treated M2 Macrophages Inhibit HCC Cell Proliferation, Migration, and EMT

**Figure 4:**
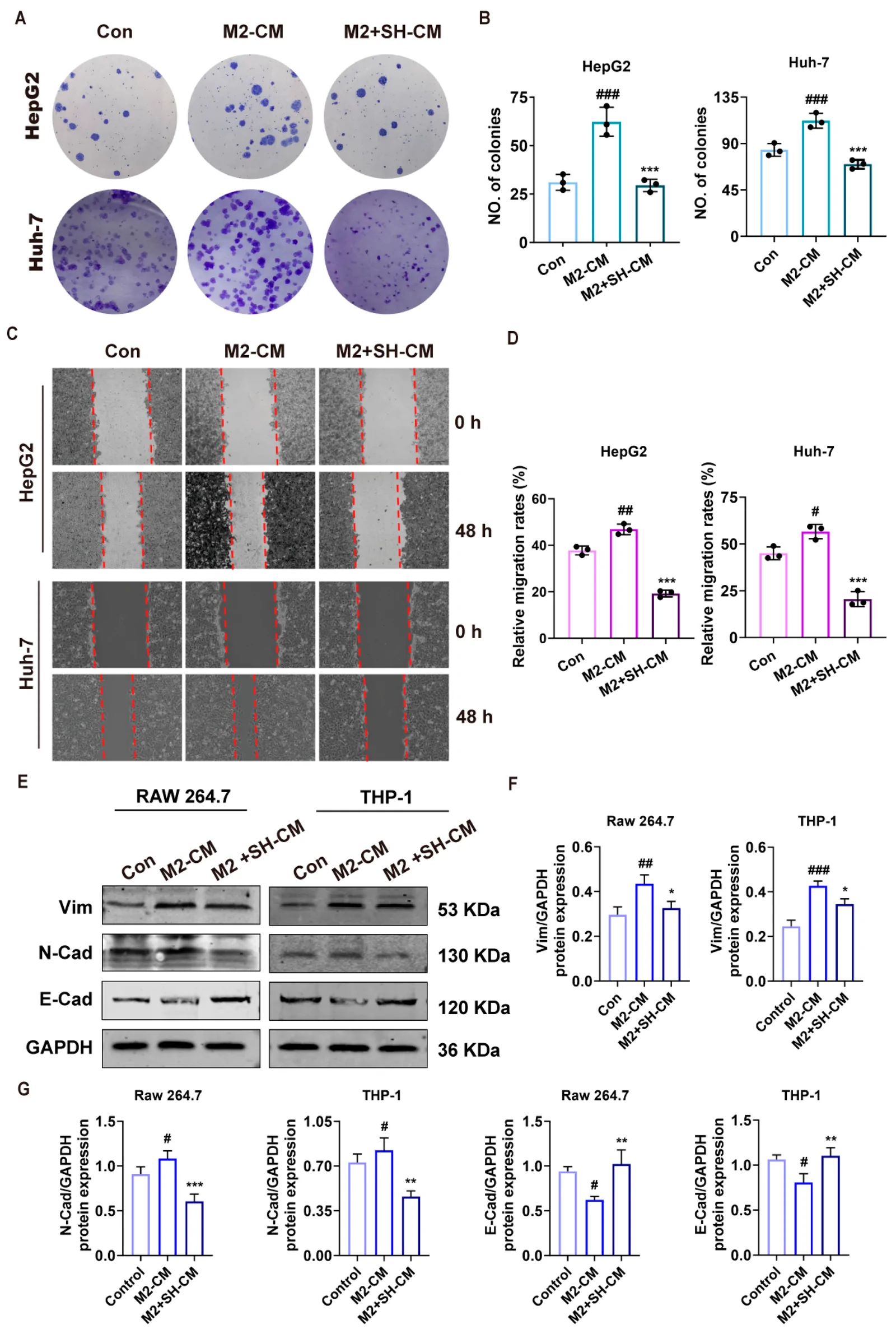
SH-treated M2 macrophages inhibit HCC cell proliferation, migration, and EMT. (**A**,**B**) Colony formation assay and quantification of HepG2 and Huh-7 cells treated with Control medium, M2-CM or M2 + SH-CM. (**C**) Representative wound-healing assay images depicting cell migration in HepG2 and Huh-7 cells at 0 and 48 h after SH treatment. (**D**) Quantification of wound healing rate. (**E**–**G**) Western blot analysis of EMT markers (Vim, N-Cad, E-Cad) in HCC cells. Data are presented as mean ± SD (*n* = 3). ### *p* < 0.001, ## *p* < 0.01 and # *p* < 0.05 versus Control; *** *p* < 0.001, ** *p* < 0.01 and * *p* < 0.05 vs. M2-CM group.

To evaluate whether SH-regulated macrophages affect the malignant behavior of HCC cells, conditioned medium was collected from M2 macrophages with or without SH treatment and applied to HepG2 and Huh-7 cells. Colony formation assays showed that M2 macrophage-conditioned medium (M2-CM) markedly increased the colony-forming ability of both HepG2 and Huh-7 cells compared with the control group. In contrast, conditioned medium from SH-treated M2 macrophages (M2 + SH-CM) significantly reduced colony formation (Figure 4A,B).

Wound-healing assays showed that M2-CM promoted HCC cell migration, as reflected by increased wound closure after 48 h. However, M2 + SH-CM significantly suppressed wound healing in both HepG2 and Huh-7 cells (Figure 4C,D). Furthermore, M2-CM increased the expression of EMT-related mesenchymal markers Vimentin and N-cadherin and reduced the epithelial marker E-cadherin. These EMT-promoting changes were reversed by M2 + SH-CM (Figure 4E–G). These results indicate that SH weakens the tumor-promoting activity of M2 macrophages and suppresses HCC cell proliferation, migration and EMT.

### 2.5. LIF-High HCC Is Characterized by M2 Macrophage Enrichment and PI3K-Akt Activation

**Figure 5:**
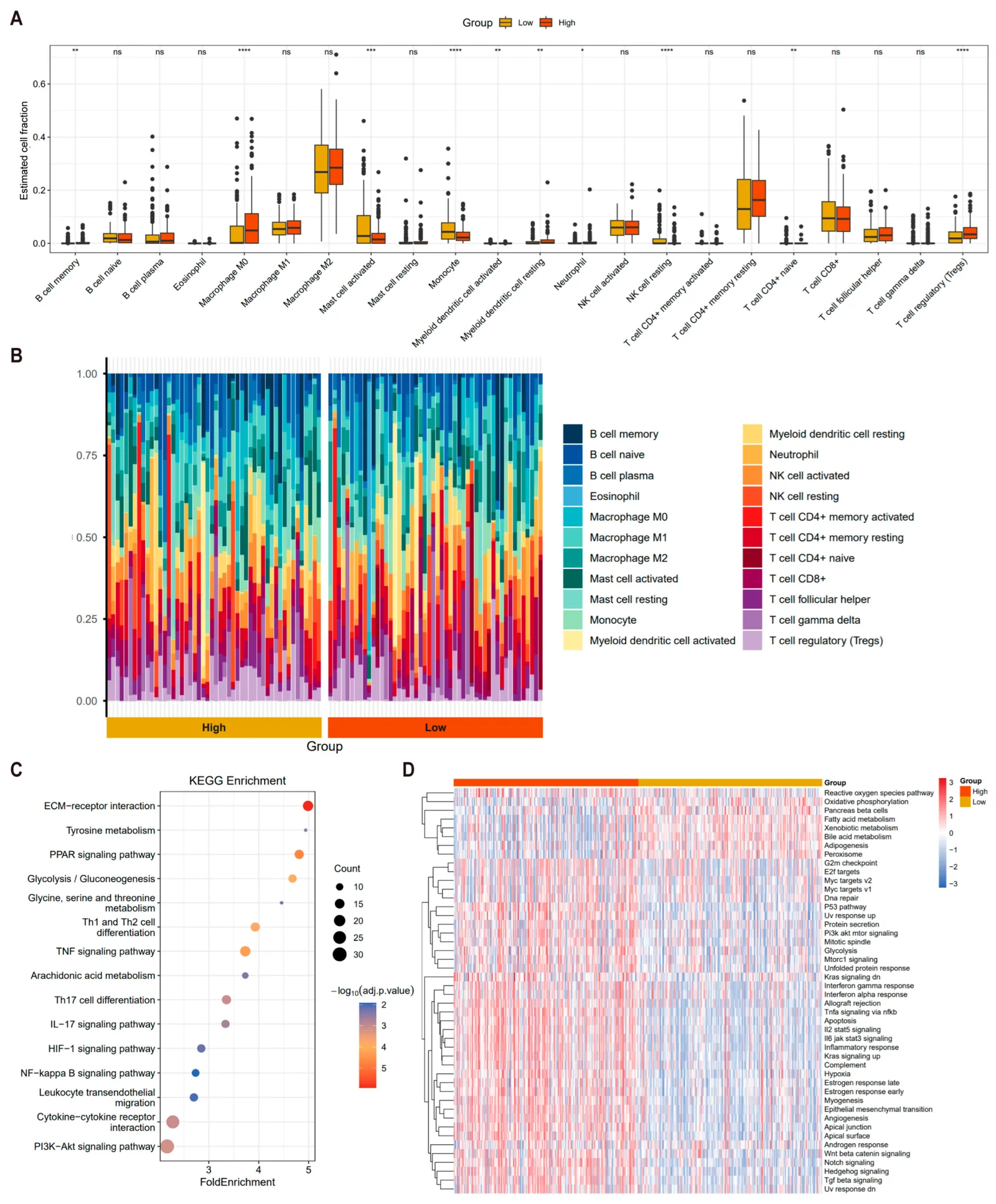
LIF-High HCC Is Characterized by M2 Macrophage Enrichment and PI3K-Akt Activation. (**A**) Boxplot showing the expression levels of infiltrating immune cell populations in LIF-high and LIF-low groups. (**B**) Stacked bar plot showing immune cell composition across LIF-high and LIF-low samples. (**C**) KEGG enrichment analysis of differentially expressed genes between LIF-high and LIF-low groups. (**D**) Heatmap showing immune- and tumor-related pathway activity between LIF-high and LIF-low groups. *ns*, not significant; **** *p* < 0.0001, *** *p* < 0.001, ** *p* < 0.01, * *p* < 0.05.

To explore the immune microenvironment driving HCC progression, we systematically performed immune infiltration analysis. Distinct immune cell distribution profiles were observed between the LIF-high and LIF-low groups based on the median LIF expression (Figure 5A,B). Specifically, the M0, M1, and M2 macrophage subsets exhibited significant intergroup differences, indicating that macrophage remodeling plays a pivotal role in HCC progression. Further bioinformatic analysis based on the TCGA-HCC dataset demonstrated that elevated M2 macrophage infiltration is closely correlated with poor clinical outcomes in HCC patients. KEGG enrichment analysis of differentially expressed genes between the LIF-high and LIF-low groups identified multiple tumor progression-related pathways significantly enriched in LIF-high HCC, including ECM–receptor interaction, PPAR signaling, glycolysis/gluconeogenesis, IL-17 signaling, and PI3K-Akt signaling, with the PI3K-Akt pathway showing the most prominent enrichment (Figure 5C). Moreover, heatmap analysis revealed distinct expression patterns of gene signatures associated with inflammatory response, epithelial–mesenchymal transition (EMT), and PI3K/Akt/mTOR signaling between the two subgroups (Figure 5D). Consistent with the KEGG results, gene set enrichment analysis (GSEA) further confirmed aberrant PI3K-Akt pathway activation in HCC samples. Collectively, these findings suggest that LIF-high HCC is characterized by M2 macrophage enrichment and PI3K-Akt activation and that M2 macrophage infiltration is associated with malignant progression and activation of PI3K-Akt, inflammatory response, EMT, and metabolic reprogramming.

### 2.6. SH Inhibits M2 Polarization via the LIF/PI3K/AKT Pathway

**Figure 6:**
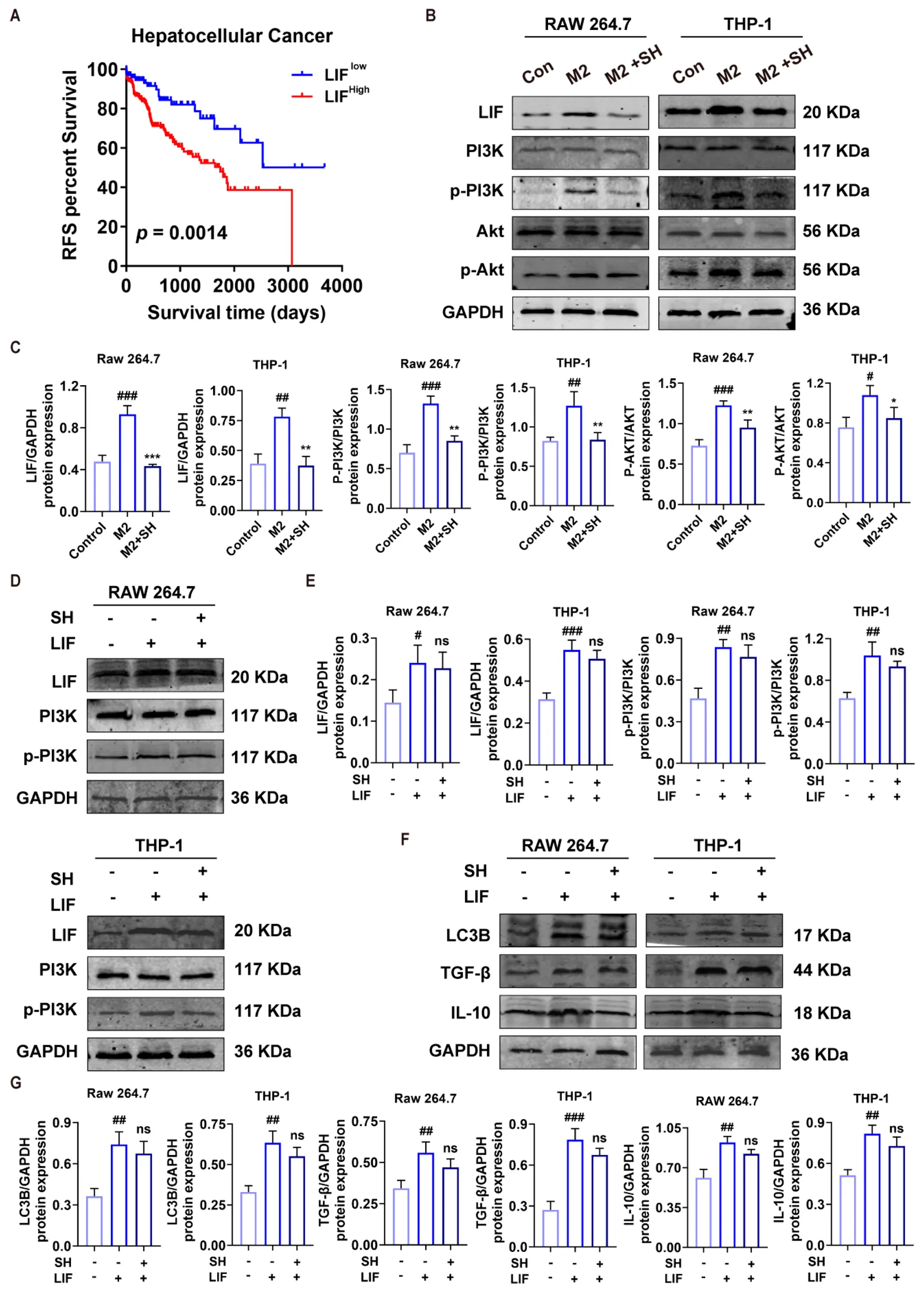
SH Inhibits M2 Polarization via the LIF/PI3K/AKT Pathway. (**A**) Kaplan−Meier analysis of recurrence−free survival (RFS) in hepatocellular carcinoma (HCC) patients stratified by LIF expression (LIF^low^ vs. LIF^high^; *p* = 0.0014). High LIF expression is associated with poorer RFS. (**B**,**C**) Western blot analysis and corresponding quantification of LIF, PI3K, p−PI3K, Akt, and p−Akt protein levels in M2-polarized RAW 264.7 and THP−1 cells with or without SH treatment. GAPDH was used as the loading control. (**D**,**E**) Western blot analysis and quantification of LIF, PI3K, and p−PI3K in RAW 264.7 and THP-1 cells under control, LIF overexpression and LIF overexpression + SH conditions. (**F**,**G**) Western blot analysis and quantification of LC3B, TGF−β and IL−10 in RAW 264.7 and THP−1 cells under the same conditions as (**D**). Data are presented as mean ± SD (*n* = 3). *ns*, not significant; ### *p* < 0.001, ## *p* < 0.01 and # *p* < 0.05 versus Control; *** *p* < 0.001, ** *p* < 0.01 and * *p* < 0.05 vs. M2 or LIF group.

In line with our earlier observations [13], LIF expression is elevated in HCC and is associated with poor clinical outcomes in HCC patients (Figure 6A). On the basis of pathway enrichment results, we further explored the LIF/PI3K/Akt signaling axis as a plausible mechanism mediating SH-regulated macrophage polarization. Western blotting showed that M2 induction increased LIF expression and activated PI3K/Akt signaling, as reflected by increased p-PI3K and p-Akt levels. SH treatment reduced LIF expression and attenuated PI3K and Akt phosphorylation in RAW264.7 and THP-1 macrophages (Figure 6B,C). To further verify the involvement of LIF signaling, macrophages were transfected with LIF-overexpressing plasmid in the presence or absence of SH. Western blot results showed that LIF overexpression showed a trend of blunting the SH-induced reduction in p-PI3K levels (Figure 6D,E). Similarly, LIF overexpression partially blunted the SH-mediated reduction in LC3B, TGF-β and IL-10 at the protein level, although these rescue comparisons did not achieve statistical significance under the current experimental conditions (Figure 6F,G). These findings suggest that SH suppresses M2 polarization at least partly through inhibition of LIF/PI3K/Akt signaling.

### 2.7. SH Suppresses HCC Growth In Vivo by Remodeling the TIME

**Figure 7:**
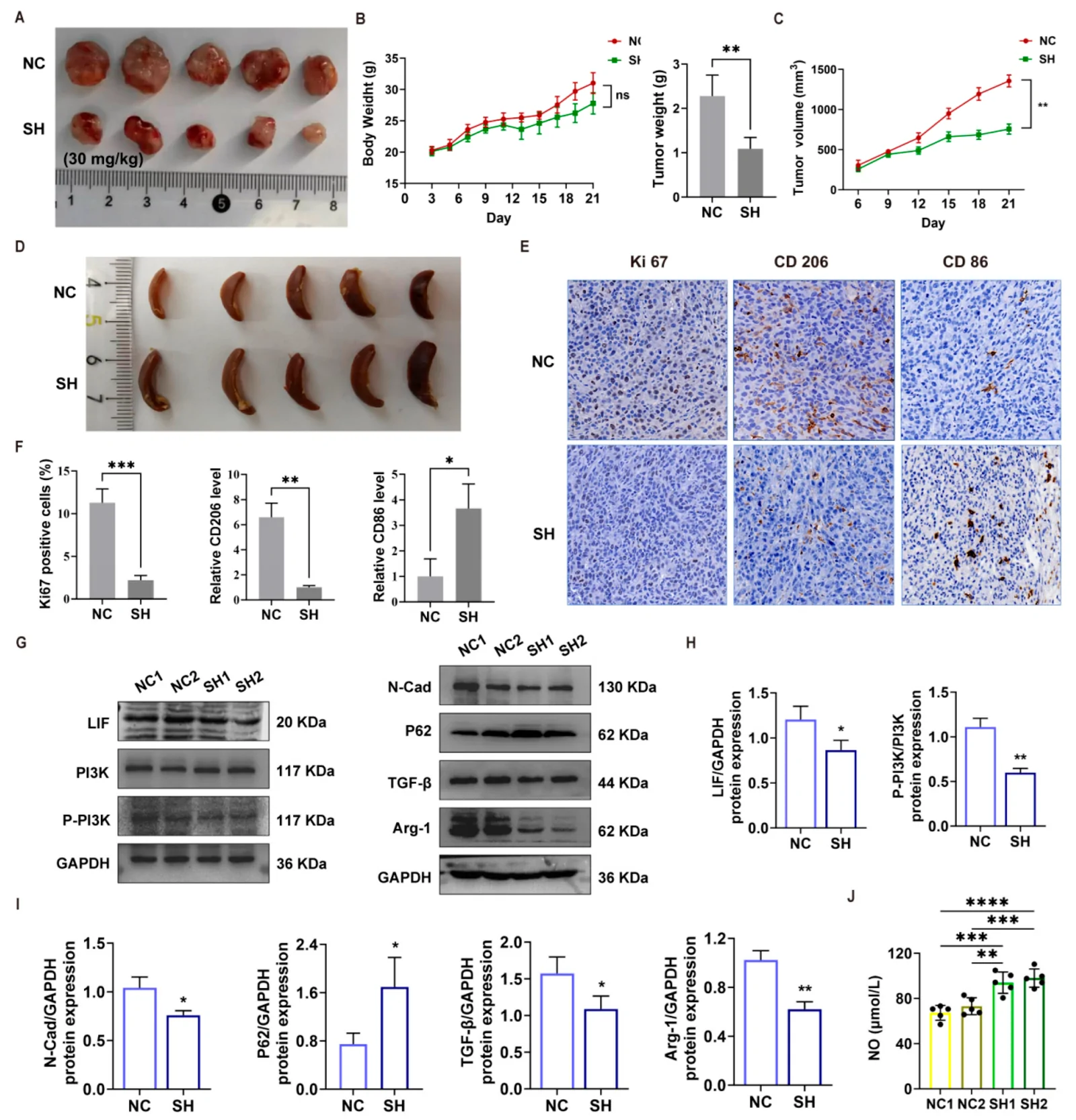
SH suppresses HCC growth in vivo by remodeling the TIME. (**A**) Representative images of subcutaneous tumors from the NC and SH (30 mg/kg) groups. (**B**) Body weight and tumor weight of mice in each group was monitored during the treatment period. (**C**) Tumor volume of the NC and SH groups. (**D**) Representative images of spleens from the NC and SH groups. (**E**) Representative immunohistochemical (IHC) staining of Ki67, CD206, and CD86 in tumor tissues from the NC and SH groups. Scale bar, 50 μm. (**F**) Quantification of Ki67-positive cells and relative expression levels of CD206 and CD86 in tumor tissues. (**G**) Western blot analysis of LIF, PI3K, P-PI3K, and GAPDH (**left**), and N-Cad, P62, TGF-β, Arg-1, and GAPDH (**right**) in tumor tissues from the NC and SH groups. (**H**) Quantitative analysis of LIF/GAPDH and P-PI3K/PI3K protein expression. (**I**) Quantitative analysis of N-Cad/GAPDH, P62/GAPDH, TGF-β/GAPDH, and Arg-1/GAPDH protein expression. (**J**) NO production in tumor tissues of the NC and SH groups. Data are presented as mean ± SD (*n* = 3). *ns*, no significant difference, * *p* < 0.05, ** *p* < 0.01, *** *p* < 0.001, **** *p* < 0.0001.

The antitumor efficacy of SH was further evaluated in tumor-bearing mice. Compared with the control group, SH treatment (30 mg/kg) significantly reduced tumor weight and tumor volume (Figure 7A–C), whereas body weight remained comparable between the two groups throughout the treatment period (Figure 7B, ns), suggesting that SH exerts antitumor effects without overt systemic toxicity. Representative images of spleens from the NC and SH groups are shown in Figure 7D. Notably, macroscopic splenic observations were made. Enlargement was observed in SH-treated tumour-bearing mice, which may be associated with SH-induced systemic immune remodelling.

Immunohistochemical (IHC) staining of tumor tissues revealed that SH markedly decreased the percentage of Ki67-positive cells (Figure 7E,F), indicating suppressed tumor cell proliferation. Notably, SH significantly reduced the expression of the M2 macrophage marker CD206 while increasing the M1 macrophage marker CD86 in the tumor microenvironment (Figure 7E,F). Consistent with these results, Western blot analysis showed that SH downregulated the M2-associated marker Arg-1, as well as LIF expression and the p-PI3K/PI3K ratio, and decreased the expression of N-Cad and TGF-β while upregulating the autophagy-associated protein P62 (Figure 7G–I). Furthermore, NO production in tumor tissues was significantly elevated following SH treatment (Figure 7J), a functional feature consistent with M1-like macrophage activation.

Collectively, these in vivo findings are consistent with the in vitro results and demonstrate that SH suppresses HCC growth by remodeling the TIME, shifting macrophage polarization from a tumor-promoting M2 phenotype toward an M1-like antitumor phenotype.

## 3. Discussion

Hepatocellular carcinoma (HCC) progression is governed not only by the intrinsic malignant properties of tumor cells but also by the immune cell landscape of the tumor microenvironment (TME). Tumor-associated macrophages (TAMs), the most abundant immune population in the TME, predominantly adopt an immunosuppressive M2 phenotype in HCC and promote tumor proliferation, migration, epithelial–mesenchymal transition (EMT), and immune evasion through the secretion of TGF-β, IL-10, and related cytokines [22,23,24]. Repolarizing TAMs from the pro-tumorigenic M2 phenotype toward the anti-tumor M1 phenotype has therefore emerged as a promising immunotherapeutic strategy for HCC [23]. Stachydrine hydrochloride (SH), an alkaloid component of *Panzeria alashanica* Kupr. (a traditional Mongolian medicinal herb widely used in Inner Mongolia), has been shown to directly suppress HCC cell proliferation and migration and to induce cellular senescence [13,25]. However, whether SH modulates the tumor immune microenvironment, particularly macrophage polarization, has remained largely unexplored. In the present study, we demonstrate that SH repolarizes macrophages from the M2 toward the M1 phenotype and thereby inhibits HCC progression, and we delineate the underlying LIF/PI3K-Akt signaling axis.

Using RAW264.7 (mouse) and THP-1 (human) macrophages, we found that SH, at a non-cytotoxic concentration (100 μmol/L), directly promoted M1 polarization, as reflected by increased expression of the M1 markers CD86 and inducible nitric oxide synthase (iNOS), elevated NO release, and upregulation of the pro-inflammatory cytokines TNF-α, COX-2, and IL-6 (Figure 1). Conversely, SH suppressed IL-4/IL-13-induced M2 polarization, downregulating CD206, TGF-β, IL-10, and Arg-1 (Figure 2). M1 macrophages exert anti-tumor cytotoxicity through the iNOS–NO axis, provide T-cell co-stimulation through CD86, and activate anti-tumor immunity through TNF-α and other inflammatory mediators, whereas M2 macrophages restrain effector T-cell function via IL-10 and TGF-β [22,26]. The capacity of SH to skew macrophage polarization toward the M1 phenotype thus constitutes the cellular basis of its anti-HCC immune effect.

At the tumor-cell level, conditioned medium from M2 macrophages (M2-CM) markedly promoted clonogenic growth, migration, and EMT (increased Vimentin and N-cadherin, decreased E-cadherin) of HepG2 and Huh-7 cells, whereas conditioned medium from SH-treated M2 macrophages (M2 + SH-CM) reversed these pro-tumorigenic effects (Figure 4). TAMs are known to induce EMT in HCC cells through TGF-β- and IL-6-driven STAT3/TGF-β-Smad signaling, a critical step in HCC invasion and metastasis [22,24]. These results indicate that SH not only directly inhibits EMT in HCC cells [25] but also indirectly abrogates TAM-mediated pro-metastatic effects by remodeling the macrophage phenotype, thereby exerting a dual anti-HCC intervention.

Mechanistically, we identified the signaling events that link SH to macrophage repolarization. M2 polarization was accompanied by elevated autophagy, as evidenced by increased ATG12 and LC3B expression and enhanced P62 degradation, consistent with reports that M2 macrophages display higher autophagic activity than M1 cells and that autophagy sustains the M2/immunosuppressive TAM phenotype [27,28]. SH treatment significantly suppressed M2-associated autophagy and, in parallel, enhanced macrophage phagocytosis of HCC cells (Figure 3), suggesting that inhibition of M2-associated autophagy contributes to M2→M1 repolarization and anti-tumor phagocytosis [27]. We also found that M2 polarization markedly upregulated the expression of leukemia inhibitory factor (LIF) and that SH concentration-dependently suppressed LIF expression and PI3K/Akt phosphorylation in M2-polarized macrophages; LIF overexpression partially blunted SH-driven phenotypic changes in macrophages at the protein level (Figure 6). Although these rescue comparisons did not reach formal statistical significance under the current experimental settings, our collective in-vitro and bioinformatic data support the LIF/PI3K-Akt axis as a plausible major signaling cascade mediating SH activity. The LIF/LIFR axis, which is overexpressed in multiple solid tumors, activates JAK/STAT3, PI3K/Akt, and mTOR downstream signaling to promote tumor progression [15], and the PI3K-Akt pathway per se is a well-established driver of M2 macrophage differentiation [29]. By linking LIF to macrophage M2 polarization within the HCC microenvironment, our findings provide a direct molecular explanation for the immunomodulatory activity of SH.

The in vivo results were highly consistent with the in vitro mechanisms. SH treatment significantly inhibited the growth of subcutaneous H22 xenograft tumors (reduced tumor volume and weight, decreased Ki67 positivity) without causing overt toxicity, as reflected by unchanged body weight (Figure 7). Intratumoral TAMs shifted from the M2 toward the M1 phenotype after SH treatment, with decreased CD206, Arg-1, and IL-10 and increased CD86, TNF-α, COX-2, and NO (Figure 7), indicating that SH remodels the tumor immune microenvironment in vivo. Consistently, bioinformatic analysis of TCGA-LIHC transcriptomic data showed that the proportion of infiltrating M2 macrophages correlated with tumor malignancy, and KEGG and gene set enrichment analyses (GSEA) identified PI3K-Akt, TNF, IL-17, NF-κB, and HIF-1 signaling and EMT, TGF-β, inflammatory response, and IL6-JAK-STAT3 gene sets as differentially enriched (Figure 5), corroborating the in vitro and in vivo findings and further supporting an immune-related anti-HCC action of SH.

The M2-suppressing activity of SH is consistent with the work of Gui et al. [30], who reported that stachydrine inhibits M2 polarization of TAMs through the JAK2/STAT3 pathway and thereby suppresses colorectal cancer liver metastasis and enhances the efficacy of anti-PD-1 immunotherapy. Our study extends this concept to the HCC context and reveals that SH achieves this effect through the LIF/PI3K-Akt axis. Taken together, these observations indicate that the M2-suppressing effect of stachydrine is broadly reproducible across tumor types, while the specific signaling pathways involved may be heterogeneous depending on the tumor microenvironment.

Stachydrine has also been widely reported to exert anti-inflammatory effects in models of sterile inflammation, such as ischemic brain injury and metabolic syndrome, downregulating TNF-α, IL-1β, and NO and suppressing NF-κB signaling [31,32]. At first glance, this appears to conflict with our observation that SH upregulates TNF-α, COX-2, iNOS, and NO in macrophages. We propose that this reflects the bidirectional immunomodulatory property of SH: under conditions of excessive inflammation, SH suppresses inflammatory responses to restore homeostasis, whereas in the immunosuppressive tumor microenvironment, SH repolarizes educated M2 macrophages toward the anti-tumor M1 phenotype. The pro-inflammatory and anti-inflammatory effects of SH are therefore not contradictory but rather represent two facets of its immune-remodeling function, the direction of which depends on the activation state of macrophages and the microenvironmental context.

From a theoretical perspective, this study identifies the LIF/PI3K-Akt signaling axis as a hub through which SH reprograms macrophage M2→M1 polarization, extending the anti-HCC mechanism of stachydrine from direct actions on tumor cells [13,25] to the remodeling of the tumor immune microenvironment, thereby defining a dual model of direct anti-tumor activity plus immunomodulation. We also systematically delineated the multi-point regulation of SH along the macrophage polarization–autophagy–phagocytosis–paracrine pro-tumorigenic signaling cascade, providing a novel framework for the tumor immunopharmacology of natural alkaloids. Combined with the finding of Gui et al. [30] that stachydrine enhances the efficacy of immune checkpoint inhibitors, and the systematic review by Tang and Huang [32] indicating that stachydrine reprograms TAMs toward an immunostimulatory phenotype, SH may serve as an adjunct for HCC immunotherapy, potentially improving responses to PD-1/PD-L1 blockade. These observations warrant further evaluation.

Several limitations of this study should be acknowledged. The in vitro experiments were restricted to the RAW264.7 (mouse) and THP-1 (human) monocyte/macrophage cell lines, and M2 polarization was induced artificially with IL-4/IL-13, which does not fully recapitulate the complex phenotypes of tumor-infiltrating macrophages in vivo; validation in human primary monocyte-derived macrophages and in TAMs isolated from HCC tissues is therefore required. The autophagy-related conclusions were based on the expression levels of LC3B, ATG12, and P62, without autophagic flux analysis (e.g., using chloroquine or bafilomycin) or pharmacological rescue experiments, so the necessity of autophagy in SH-mediated macrophage repolarization is not yet fully established. In addition, our phagocytosis assays were conducted exclusively using HepG2 HCC cells. Additional experiments across multiple HCC cell lines are needed to verify whether the SH-mediated enhancement in macrophage phagocytosis against HCC cells is a generalizable phenomenon. Importantly, our current LIF overexpression transfection experiments lack critical comparator groups, including empty-vector controls, SH-only treatment, and M2-polarized conditions; therefore, a formal rescue relationship cannot be established from these data. Follow-up rescue experiments incorporating a full panel of experimental groups will be necessary to validate the causal involvement of the LIF/PI3K/Akt axis. The mouse subcutaneous tumor model does not completely mimic the hepatic microenvironment, immune cell infiltration, and metastasis of HCC, and orthotopic or metastatic models are lacking.

Future work should validate the TAM-reprogramming activity of SH using human primary macrophages, liver cancer organoids, and orthotopic or patient-derived xenograft models; establish the causal role of autophagy using autophagic flux assays and pharmacological modulators; and systematically assess the combination of SH with immune checkpoint inhibitors, together with pharmacokinetic and long-term safety studies, to advance clinical translation.

## 4. Materials and Methods

### 4.1. Cell Lines and Reagents

RAW 264.7 (mouse macrophage), THP-1 (human monocyte), H22 (mouse hepatoma cell), HepG2, and Huh-7 (human HCC) cell lines were obtained from the Cell Bank of the Chinese Academy of Sciences. Stachydrine hydrochloride was purchased from Shanghai yuanye Bio-Technology Co., Ltd., Shanghai, China (CAS No. 4136-37-2; Purity > 98%). Primary antibodies against TGF-β (1:1000, K107847P), IL-10 (1:1000, K100332P), Arg-1 (1:1000, K106497P), ATG12 (1:1000, K105744P), LC3B (1:1000, K008014P), and p62 (1:1000, K005444P) were purchased from Solarbio (Beijing, China). Primary antibodies against TNF-α (1:1000, ab34674), COX-2 (1:1000, ab15191), IL-6 (1:1000, ab290735), iNOS (1:1000, ab178945), LIF (1:1000, ab138002) and Ki-67 (ab16667) were purchased from Abcam (Cambridge, UK). Primary antibodies against total AKT (1:1000, 10176-2-AP), p-AKT (1:2000, Ser473, 66444-1-Ig), Vimentin (1:3000, 80232-1-RR), N-cadherin (1:2000, 66219-1-Ig), E-cadherin (1:1000, 20874-1-AP) and GAPDH (1:5000, 60004-1-Ig) were purchased from Proteintech (Wuhan, China). Primary antibodies against PI3K (bs-2067R, 1:1000) and p-PI3K (1:1000, Tyr317, bs-5570R) were purchased from Beijing Biosynthesis Biotechnology Co. (Beijing, China). Fluorochrome-conjugated antibodies against F4/80 (111704, Alexa Fluor 488), CD86 (333807) and CD206 (141703) were obtained from BioLegend (San Diego, CA, USA). Human/mouse IL-4 recombinant protein (200-04-5UG, 214-14-5UG), human/mouse IL-13 recombinant protein (200-13-10UG, 210-13-10UG), and human/mouse IFN-γ recombinant protein (300-02-100UG, 315-05-100UG) were purchased from Invitrogen (Thermo Fisher Scientific, Waltham, MA, USA).

### 4.2. Cell Viability Assay

RAW 264.7 cells were digested, counted and seeded at 2 × 10^4^ cells/well in 96-well plates and cultured overnight for attachment. Suspended THP-1 monocytes were differentiated into adherent macrophages using PMA (100 ng/mL, 24 h). After stabilisation, cells were exposed to serial concentrations of SH for 48 h. MTT stock solution (5 mg/mL, 20 μL per well) was added, followed by 4 h incubation for formazan formation. Supernatants were gently removed, and 100 μL DMSO was added. Plates were shaken at low speed to dissolve crystals, and absorbance was detected at 490 nm on a microplate reader. Three technical replicates were used for each condition, and the experiment was repeated three independent times.

### 4.3. Western Blotting

Total protein was extracted from cells or tumor tissues. After cell or tissue lysis using RIPA buffer, total protein samples were subjected to SDS-PAGE electrophoresis and subsequently transferred onto polyvinylidene difluoride (PVDF, Millipore, Billerica, MA, USA) membranes. Then the membranes were incubated with antibodies against TNF-α, COX-2, IL-6, iNOS, TGF-β, IL-10, Arg-1, ATG12, LC3B, P62, LIF, PI3K, p-PI3K, Akt, p-Akt, Vimentin, N-cadherin, E-cadherin and GAPDH. Western blot bands were scanned and quantified using the Odyssey CLx near-infrared dual-color laser imaging system (LI-COR Biosciences, Lincoln, NE, USA).

### 4.4. Flow Cytometry

CD86 and CD206 were used to evaluate M1 and M2 macrophage populations, respectively. M1 macrophage polarization was induced by treatment with LPS (100 ng/mL) plus IFN-γ (20 ng/mL) for 48 h. For M2 polarization, cells were treated with IL-4 (20 ng/mL) and IL-13 (20 ng/mL) for 48 h.

Huh-7 cells were stably labelled with GFP, and drug (100 μM) treatment was conducted for 48 h. Macrophages receiving indicated treatments were co-cultured with GFP-positive Huh-7 cells at an effector-to-target ratio of 1:1 for 12 h. Macrophages were then harvested, and the proportion of macrophages engulfing GFP-labelled HCC cells was quantified using a CytoFLEX flow cytometer (Beckman Coulter, Inc., Brea, CA, USA). The phagocytic percentage corresponds to the fraction of GFP-positive macrophages within the total macrophage population.

### 4.5. Macrophage Polarization

Macrophage Polarization RAW 264.7 mouse macrophages and PMA-differentiated THP-1-derived human macrophages were polarized toward the M0, M1, or M2 phenotype under defined in-vitro conditions. The M1 phenotype was induced by combined stimulation with lipopolysaccharide (LPS) and interferon-γ (IFN-γ), whereas M2-type polarization was triggered via interleukin-4 (IL-4) plus interleukin-13 (IL-13) treatment. Parallel groups received the above polarization stimuli together with 100 μM SH for intervention. Cellular morphological alterations under distinct treatment conditions were documented by bright-field light microscopy. To characterize macrophage polarization status, the expression signature of representative M1- and M2-related markers was assessed using multiple complementary approaches, including Western blot analysis, quantitative real-time polymerase chain reaction (qRT-PCR), and flow cytometry.

### 4.6. Immunofluorescence

Immunofluorescence targeting LC3B was performed to assess autophagic activity in macrophages. Briefly, cells were washed with pre-chilled PBS and fixed in 4% paraformaldehyde for 10 min. Permeabilization was carried out using 0.5% Triton X-100 (Thermo Fisher Scientific, USA). After permeabilization, samples were blocked with bovine serum albumin (BSA) to minimise non-specific antibody binding, followed by overnight incubation with primary antibodies at 4 °C. On the following day, samples were incubated with fluorophore-conjugated secondary antibodies for 1 h at ambient room temperature. Cell nuclei were counter-stained with DAPI. Fluorescent images were captured under an inverted fluorescence microscope (Carl Zeiss, Oberkochen, Germany), and fluorescence intensity of LC3B was quantitatively analysed using ImageJ software (1.48v).

### 4.7. Wound Healing Assay

Macrophage-derived conditioned medium from distinct treatment groups was harvested and subsequently used to treat HepG2 and Huh-7 hepatocellular carcinoma cells. Colony-formation and wound-healing scratch assays were implemented to assess tumour-cell proliferative capacity and migratory potential, respectively. For the wound-healing scratch assay, HepG2 and Huh-7 cells were seeded into six-well culture plates and grown to full confluence. A sterile 200 μL pipette tip was utilised to create uniform linear scratches across the cell monolayer. Detached cellular debris was removed by gentle washing, and fresh macrophage-conditioned medium was replenished. Bright-field images of the scratch wounds were documented at 0 h and 48 h post-scratching for migratory-capacity analysis. The relative cell migration rate was calculated according to the formula: (initial scratch area − residual scratch area)/initial scratch area × 100%.

### 4.8. Plasmids and Transfection

RAW 264.7 mouse macrophages and PMA-differentiated THP-1-derived human macrophages were used. We designed and synthesized LIF overexpression plasmid (HanBio Technology, Shanghai, China), with the following sequences: CMV-F: 5′-CGCAAATGGGCGGTAGGCGTG-3′ and pEGFP-3: 5′-CGTCGCCGTCCAGCTCCAGCTCGACCAG-3′. For each well of 6-well plates, 2 μg of LIF-overexpressing plasmid was mixed with 6 μL Lipofectamine 2000 (Invitrogen, Thermo Fisher Scientific, Waltham, MA, USA) according to manufacturer protocols. Transfection was performed for 24 h, and then cells were treated with 100 μM SH for an additional 48 h before cell lysis for Western blot analysis.

### 4.9. Bioinformatic Analyses

Transcriptomic data of 367 hepatocellular carcinoma tumor samples were downloaded from the TCGA database (https://portal.gdc.cancer.gov/, accessed on 2 June 2025). Relative fractions of 22 immune-cell subsets were calculated by CIBERSORT (PMID: 25822800) using a 547-gene signature matrix; the sum of estimated immune-cell proportions per sample was 1. Samples were stratified into LIF-high and LIF-low groups based on the median LIF expression (log2 (TPM + 1) = 1.2739). Immune-cell infiltration scores were compared between groups (Figure 5A,B). Differentially expressed genes (DEGs) were screened using the R package limma (v3.62.2, PMID: 25605792). The Benjamini–Hochberg (BH) method was used for multiple-testing correction. DEG thresholds were set as │Log2FC│ > 1 and FDR < 0.05. KEGG pathway enrichment for DEGs was performed with clusterProfiler (v4.14.6, PMID: 22455463). BH correction was applied, and pathways with an adj.*p*.value < 0.05 were considered significant. Fifteen significant pathways were visualized in a bubble plot (Figure 5C). GSVA (v2.0.7, PMID: 23323831) was used to compute HALLMARK pathway enrichment scores for each sample. Differential pathways between LIF subgroups were identified via limma with BH correction, using an adj.*p*.value < 0.05 as the cutoff. A heatmap was plotted to exhibit scores of differential pathways (Figure 5D). Recurrence-free survival (RFS) was defined as the time from surgery to tumor recurrence or death from any cause, whichever occurred first; patients without an event were censored at the last follow-up. Kaplan–Meier curves were plotted, and the log-rank test was used to compare RFS between LIF-high and LIF-low subgroups in the GSE54236 hepatocellular carcinoma cohort (https://www.ncbi.nlm.nih.gov/geo/query/acc.cgi?acc=GSE54236, accessed on 10 June 2025). Subgroups were stratified by the median LIF expression level (Figure 6A).

### 4.10. In Vivo Tumor Experiment

Male C57BL/6 mice (6–8 weeks, 18–22 g) were procured from the Animal Centre of Inner Mongolia Medical University and used for tumour inoculation. They were housed under controlled environmental conditions with maintained temperature and relative humidity (45–55%), a standard 12 h light–dark cycle, and free access to sterilised food and drinking water. Under aseptic conditions, each mouse received a subcutaneous injection of 100 μL of H22 cell suspension (containing 1 × 10^6^ viable tumour cells) into the right flank. Once palpable subcutaneous tumours had developed (approximately 80–100 mm^3^), tumor-bearing mice received intraperitoneal injection of SH (30 mg/kg/day) or vehicle control for 21 consecutive days. Body weight and tumor volume were monitored every 3 days throughout treatment. At the experimental endpoint, mice were euthanized, and tumor tissues as well as spleens were harvested for subsequent Western blot and immunohistochemical analyses. All animal care and experimental procedures adhered to ethical guidelines approved by the Inner Mongolia Medical University Animal Centre and Use Committee (approval number: YKD202403045). All animal manipulations complied with ARRIVE guidelines.

### 4.11. Immunohistochemistry Analysis

Formalin-fixed paraffin-embedded (FFPE) tissue sections (4 μm thickness); xylene; gradient ethanol (100%, 95%, 85%, 70%); phosphate-buffered saline (PBS, pH 7.4); citrate antigen retrieval buffer (0.01 M, pH 6.0); 3% hydrogen peroxide solution; normal goat serum; primary antibodies against Ki-67, CD86 and CD206; horseradish peroxidase (HRP)-conjugated secondary antibody; 3,3′-diaminobenzidine (DAB) chromogenic substrate; hematoxylin; and neutral balsam were used. All antibodies were validated for FFPE immunohistochemistry (IHC) application and used according to the manufacturer’s instructions.

### 4.12. Statistical Analysis

GraphPad Prism software (version 8.0.2) was used for data analysis. Statistically significant differences were determined by Student’s *t*-test (unpaired, two-tailed) for two groups or one-way ANOVA for multi-group samples. The data were expressed as the mean ± SD, and *p* < 0.05 was considered statistically significant.

## 5. Conclusions

In conclusion, SH suppresses HCC progression by reprogramming macrophage polarization. SH promotes M1-like activation, enhances CD86 expression, increases NO production and strengthens macrophage phagocytosis of HCC cells. Meanwhile, SH inhibits M2 polarization by downregulating TGF-β, IL-10, Arg-1 and CD206. Mechanistically, SH inhibits M2-associated autophagy and attenuates the LIF/PI3K/Akt signaling pathway. However, because the rescue experiments did not reach statistical significance, the causal requirement of this pathway remains to be established. Functionally, SH-treated macrophages reduce HCC cell proliferation, migration and EMT, and SH suppresses tumor growth in vivo while remodeling tumor-infiltrating macrophages toward an anti-tumor phenotype. These findings suggest that SH is a promising candidate for HCC immunomodulatory therapy targeting macrophage polarization.

## Data Availability

The data presented in this study are available on request from the corresponding authors due to privacy.
